# Hippocampal calcification on brain CT: prevalence and risk factors in a cerebrovascular cohort

**DOI:** 10.1007/s00330-018-5372-8

**Published:** 2018-04-04

**Authors:** Remko Kockelkoren, Jill B. De Vis, M. Stavenga, Willem P.Th.M. Mali, Jeroen Hendrikse, Annemieke M. Rozemuller, Huiberdina L. Koek, Irene C. van der Schaaf, Birgitta K. Velthuis, Pim A. de Jong, the DUST study group

**Affiliations:** 1 Department of Radiology, University Medical Center, Room E01.132, PO Box 85500, 3508 GA Utrecht, The Netherlands; 2 Department of Radiology, Johns Hopkins Medical Center, Baltimore, MD USA; 3 Department of Pathology, University Medical Center, Utrecht, The Netherlands; 4 Department of Geriatrics, University Medical Center, Utrecht, The Netherlands

**Keywords:** Hippocampus, Vascular calcification, Risk factors, Prevalence, Stroke

## Abstract

**Objectives:**

Recently, hippocampal calcification as observed on brain CT examinations was identified in over 20% of people over 50 years of age and a relation between hippocampal calcification and cognitive decline was shown. We determined the prevalence and investigated the vascular risk factors of hippocampal calcification in patients with cerebrovascular disease.

**Methods:**

Hippocampal calcification was scored bilaterally on presence and severity on CT examinations in a cohort of 1130 patients with (suspected) acute ischaemic stroke. Multivariable logistic regression analysis, adjusting for age and gender as well as adjusting for multiple cardiovascular disease risk factors, was used to determine risk factors for hippocampal calcification.

**Results:**

Hippocampal calcification was present in 381 (34%) patients. Prevalence increased with age from 8% below 40 to 45% at 80 years and older. In multivariable logistic regression analysis, age per decile (OR 1.41 [95% CI 1.26–1.57], *p* < 0.01), hypertension (OR 0.74 [95% CI 0.56–0.99], *p* = 0.049), diabetes mellitus (OR 1.57 [95% CI 1.10–2.25], *p* = 0.01) and hyperlipidaemia (OR 1.63 [95% CI 1.20–2.22], *p* < 0.01) were significantly associated with hippocampal calcification.

**Conclusions:**

Hippocampal calcification was a frequent finding on CT in this cohort of stroke patients and was independently positively associated with hyperlipidaemia and diabetes mellitus, suggesting an atherosclerotic origin.

**Key Points:**

*• Hippocampal calcification is prevalent in over 30% of cerebrovascular disease patients.*

*• Prevalence increases from 8% below 40 to 45% over 80 years.*

*• Hippocampal calcification is associated with cardiovascular risk factors hyperlipidaemia and diabetes mellitus.*

**Electronic supplementary material:**

The online version of this article (10.1007/s00330-018-5372-8) contains supplementary material, which is available to authorized users.

## Introduction

Hippocampal calcification (HC) has only recently been described in vivo for the first time using computed tomography (CT) and was found to be surprisingly common, occurring in up to 20% of subjects over 50 years of age [1]. These calcifications could not be differentiated from the innocuous plexus choroideus calcifications, however, recent advances in CT equipment and reconstruction protocols enabled the differentiation between these two.

To our knowledge just one study on the histology of the hippocampus described calcification of hippocampal vessels and called it vascular fibrosis and calcification (VFC) [2]. In this study, VFC was found to be mostly located in the precapillary and capillary arteries in the dentate gyrus and Cornu Ammonis 1 (CA1) of the hippocampal tail and body [2]. On CT examinations HC was observed in the same locations, suggesting a similar origin [1, 3]. In the pathology specimens, especially severe HC co-localized with areas of hippocampal neural loss and atrophy and it was suggested that this was due to hypoperfusion because of vascular disease [2].

In a preliminary study it was shown that HC, as measured with CT, was more common in patients with cognitive problems compared to those without [3]. Although currently still speculative, it may be that HC and vascular disease in the tail and body of the hippocampus play a role in cognitive decline and dementia. We were interested in the relation of HC to (potentially modifiable) classic risk factors for vascular disease as they could provide insight into the underlying pathological mechanism of HC and possibly prevention or treatment options. Therefore, given the potential relevance and the limited knowledge on the underlying aetiology of HC, we aimed to determine the relation between HC and classic risk factors of vascular disease in a cohort with a high prevalence of vascular risk factors.

## Methods

### Population

The study population consisted of 1393 patients who were included in the Dutch acute stroke trial (DUST), a prospective multicentre cohort study of (suspected) acute ischaemic stroke patients. The DUST study protocol was published previously [4]. Patients were included from May 2009 until July 2013 in one of six university hospitals and eight non-university hospitals in the Netherlands. All patients over 18 years of age with symptoms of acute ischaemic stroke of less than 9-h duration were included. All patients underwent non-contrast-enhanced CT, CT angiography and CT perfusion examinations within 9 h of the start of the symptoms. Patients with known contrast allergy or renal failure were excluded. Specifically for this study we excluded patients for whom no good quality (thin-slice) unenhanced CT was available (*n* = 263). Ethical approval was obtained from the medical ethics committee of the University Medical Center Utrecht, the Netherlands, as well as the local medical ethics committees of the participating centres. All patients or their legal representatives signed informed consent. If a patient died before consent could be obtained, the need for consent was waived by the medical ethics committee.

### Baseline measurements

After inclusion, additional patient data were obtained, including age, gender, a medical history of stroke, history of hypertension (systolic blood pressure ≥ 140 mmHg and/or a diastolic blood pressure ≥ 90 mmHg), diabetes mellitus (DM), hyperlipidaemia, first-degree family history of cardiovascular disease (CVD) (< 60 years of age), smoking (current, former or never), body mass index (BMI) and estimated glomerular filtration rate (eGFR) calculated using the Modification of Diet in Renal Disease (MDRD) formula in micromoles per litre. An important factor in calculating the eGFR using MDRD is race. As no information on race was available in this cohort and with the majority of the population in the Netherlands being Caucasian, eGFR was calculated assuming all patients were non-black. Analyses were repeated under the assumption that all patients were black but this did not change the results of the analyses. For our analysis, we looked at eGFR and BMI dichotomously, with a threshold of 60 μmol/L for eGFR and 30 for BMI.

### Technical information

Multidetector row CT scanners were used, with the number of detectors ranging from 40 to 320 (LightSpeed VCT, GE Healthcare, Milwaukee, Wisconsin; Brilliance 40, Brilliance 64 and Brilliance iCT 256, Philips Healthcare, Best, the Netherlands; Sensation 64, Siemens, Erlangen, Germany; Aquilion ONE, Toshiba Medical Systems, Tokyo, Japan) at 120 kV and 300–375 mAs using a standard convolution kernel and an image matrix of 512 × 512. Patients were scanned from the skull base to the vertex and scans were reconstructed using filtered back projection (FBP) with a slice thickness ranging from 0.625 to 1 mm.

### Calcification measurements

**Fig. 1:**
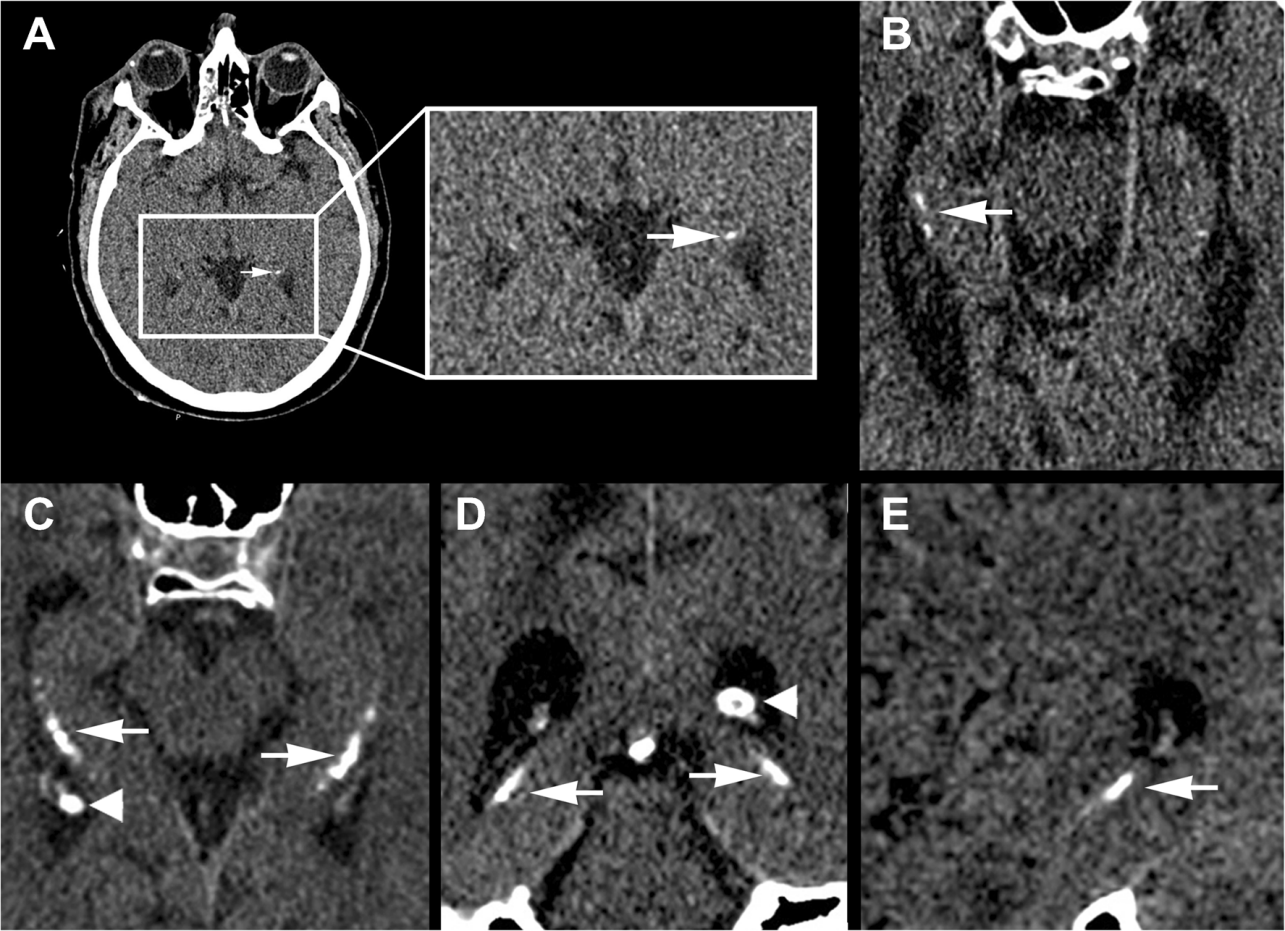
Hippocampal calcification severity. The severity of hippocampal calcifications (arrows) was scored separately in both the left and right hippocampus as **a** mild (one dot), **b** moderate (multiple dots) and **c**–**e** severe (confluent calcifications). Calcifications were scored on axial (**a**–**c**), coronal (**d**) and sagittal (**e**) reconstructions. Calcification of the choroid plexus was also visible (arrowheads)

The non-contrast-enhanced thin slice reconstructions were rated blinded and individually by two experienced radiologists and a medical practitioner with 4, 14 and 2 years of experience in reading (brain) CT scans (J.B.D.V, P.A.d.J and R.K. respectively). The cohort was divided into equal parts among the three observers. HC is seen in the brain CT window setting (centre 40, width 100) as (clustered) dense configurations comparable to bone located in the hippocampus. HC was scored bilaterally in the hippocampus as absent, mild (one dot), moderate (multiple dots) or severe (confluent) (Fig. 1) [3]. Thin slice reconstructions were used to detect subtle calcifications as they can be easily missed on 5-mm-thick slice images. To allow for better distinction of mild HC and the increased image noise present on thin slice images, HC was only scored if the calcification was present on two adjacent axial image slices. An example of image noise and mild HC, as scored in this study, is added in the online supplement (Supplementary Fig. I). Additionally, images were analysed in the coronal and sagittal plane to establish whether a calcification was located within the hippocampus or choroid plexus. The most severe calcification of either side was used to analyse HC severity.

### Statistical analysis

Descriptive statistics were used to describe the characteristics of the study population; means with standard deviations and medians with interquartile ranges (IQR: Q1, Q3) for continuous variables, depending on their distribution, and counts and percentages for categorical variables. To determine differences in baseline values between groups (HC present/absent and severity) chi-squared, Mann–Whitney *U* and analysis of variance (ANOVA) tests were used for categorical and continuous (parametric/nonparametric) data respectively.

The relation between HC presence and risk factors was studied in multivariable logistic regression analysis. First, we looked at the crude or unadjusted relation between HC and the individual risk factors. Second, we constructed a multivariable model in which we adjusted for age and gender. Lastly, we constructed a multivariable model in which we adjusted for all cardiovascular risk factors: age, gender, history of stroke, hypertension, diabetes mellitus, hyperlipidaemia, smoking (current, former, never), BMI (< 30 or ≥ 30), eGFR (< 60 or ≥ 60) and family history of CVD below the age of 60. Multiple imputation using the Markov chain Monte Carlo method was applied to complete the data set in case of missing variables (Table 1).

**Table 1:** Baseline characteristics by hippocampal calcification presence

Characteristic	Total, *n* = 1130 (100%)	HC present, *n* = 381 (33.7%)	HC absent, *n* = 749 (66.3%)	*p* value	Missing %
Age, years (median, IQR)	68.7 (20)	72.7 (16)	67.2 (21)	< .01	0
Gender (male)	643 (56.9)	217 (57.0)	426 (56.9)	.98	0
Stroke in medical history	276 (24.4)	114 (29.9)	162 (21.6)	< .01	0.6
Hypertension	596 (52.7)	208 (54.6)	388 (51.8)	.35	0.9
Diabetes mellitus	171 (15.1)	79 (20.7)	92 (12.3)	< .01	0.4
Hyperlipidaemia	388 (35.3)	165 (45.1)	223 (30.5)	< .01	2.8
Smoking				.43	7
Current	303 (28.8)	102 (29.1)	201 (28.7)		
Former	346 (32.9)	123 (35.1)	223 (31.8)		
Never	352 (38.2)	125 (35.7)	227 (39.5)		
Obesity, BMI > 30	146 (20.6)	48 (19.5)	98 (21.1)	.61	37
eGFR < 60	166 (14.6)	54 (14.2)	112 (15.0)	.85	0.7
1st degree family < 60 years with CVD history	245 (32.8)	77 (33.3)	168 (32.6)	.85	34

*HC* hippocampal calcification, *IQR* interquartile range, *CVD* cardiovascular disease, *eGFR* estimated glomerular filtration rate [ml/min/1.73 m^2^, = 32,788 × (serum creatinine)^−1.154^ × (age)^−0.203^ × (0.742 if woman) × (1.210 if black)], *BMI* body mass index in kg/m^2^, data presented as *n* (%)

Interobserver agreement of HC presence (yes/no) and the HC severity score (absent, mild, moderate, severe) was calculated between all three observers in a sample of 50 scans (100 hippocampi) from the cohort using a Fleiss’ kappa and a weighted kappa (squared) respectively. These 50 scans were randomly distributed through each observer’s cohort. A consensus reading determined the final score if there were any discrepancies between the three observers.

Statistical significance was defined as *p* < 0.05. Statistical analysis was performed using SPSS (IBM SPSS Statistics, Version 23.0. IBM Corp, Armonk, NY) and R (R Foundation for Statistical Computing, Vienna, Austria. https://www.R-project.org/).

## Results

Characteristics of the study population can be found in Table 1. The final cohort consisted of 1130 patients whose median age was 68.7 years (IQR 48.7–88.7) and 643 (56.9%) were male. A history of hypertension (53%) and hyperlipidaemia (35%) and a family history of CVD (33%) were highly prevalent. Patients with HC were, on average, older and more often had a history of stroke, DM and hyperlipidaemia.

### Hippocampal calcification

Interobserver agreement was good with a Fleiss’ kappa of 0.81 (95% CI 0.70–0.92) for HC presence and weighted kappa statistics of 0.91, 0.88 and 0.93 for HC severity between the three observers.

**Fig. 2:**
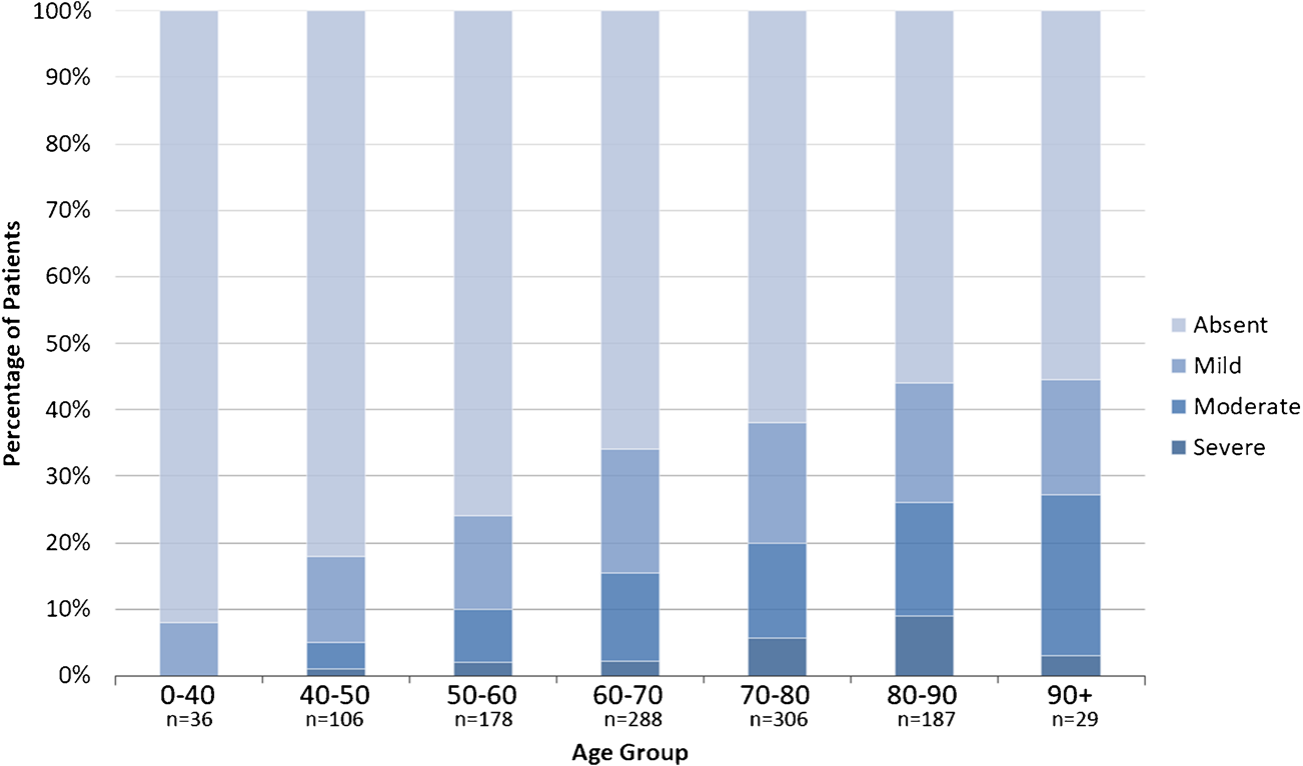
Hippocampal calcification severity per age group (*n* = number)

HC was present in 381 (34%) patients and was mild in 188 (17%), moderate in 139 (12%) and severe in 54 (5%). Patient characteristics per severity group can be found in Table 2. With increasing severity patients were on average older and more frequently had a history of stroke, DM and hyperlipidaemia. Severity per age group is depicted in Fig. 2 and shows an increase in percentage of moderate and severe HC with increasing age. Prevalence increased with age from 8% in patients younger than 40 years of age to 45% at 80 years and older. HC was found most frequently bilaterally 223 (58%), followed by unilaterally right 102 (27%) and left 56 (15%) (Table 3).

**Table 2:** Severity of hippocampal calcification in relation to risk factors

Determinant	Absent, *n* = 749	Mild, *n* = 188	Moderate, *n* = 139	Severe, *n* = 54	*p* value
Age, years (median, IQR)	67.2 (21)	69.6 (17)	70.3 (15)	74.0 (17)	< .01
Gender (male)	426 (56.8)	109 (58.3)	76 (54.7)	32 (59.3)	.92
Stroke in medical history	162 (21.6)	53 (28.3)	40 (29.2)	21 (38.9)	< .01
Hypertension	388 (51.7)	99 (53.2)	77 (55.8)	32 (61.5)	.548
Diabetes mellitus	92 (12.3)	36 (19.3)	32 (23.3)	11 (20.4)	< .01
Hyperlipidaemia	223 (30.5)	83 (45.1)	57 (43.5)	25 (49.0)	< .01
Smoking					.22
Current	201 (28.7)	43 (25.1)	38 (29.5)	21 (42.9)	
Former	223 (31.8)	66 (38.4)	42 (32.6)	15 (30.6)	
Never	227 (39.5)	62 (36.6)	49 (38.0)	13 (26.5)	
Obesity, BMI > 30	98 (21.1)	32 (24.4)	14 (17.7)	2 (5.6)	.08
eGFR < 60	112 (15.0)	20 (14.5)	9 (16.7)	39 (15.2)	.94
1st degree family < 60 years with CVD history	168 (32.6)	32 (27.1)	37 (42.0)	8 (32.0)	.16

*IQR* interquartile range, *CVD* cardiovascular disease, *eGFR* estimated glomerular filtration rate [ml/min/1.73 m^2^, = 32,788 × (serum creatinine)^−1.154^ × (age)^−0.203^ × (0.742 if woman) × (1.210 if black)], *BMI* body mass index in kg/m^2^, data presented as *n* (%)

**Table 3:** Hippocampal calcification severity in the left and right hippocampus

		HC severity right				
		Absent	Mild	Moderate	Severe	Total
HC severity left	Absent	749	80	19	3	l851
	Mild	42	66	31	4	143
	Moderate	11	13	65	12	101
	Severe	3	3	3	26	35
Total		805	162	118	45	1130

*HC* hippocampal calcification

### Uni- and multivariable regression analysis

Results of the uni- and multivariable logistic regression analysis are presented in Table 4. After adjusting for age and gender, age per decile (OR 1.37, 95% CI 1.24–1.51, *p* < 0.01, adjusted for gender), history of stroke (OR 1.40, 95% CI 1.08–1.87, *p* = 0.02), DM (OR 1.68, 95% CI 1.20–2.35, *p* < 0.01) and hyperlipidaemia (OR 1.71, 95% CI 1.31–2.22, *p* < 0.01) were significant. When adjusting for all CVD risk factors, age per decile (OR 1.40, 95% CI 1.26–1.57, *p* < 0.01), hypertension (OR 0.74, 95% CI 0.56–0.99, *p* = 0.049), DM (OR 1.57, 95% CI 1.10–2.25, *p* = 0.01) and hyperlipidaemia (OR 1.63, 95% CI 1.20–2.22, *p* < 0.01) were significant. Additionally, because of the borderline significant OR < 1 for hypertension, the effect of treatment with antihypertensive drugs in patients with a history of hypertension was assessed. Treated hypertension remained significant (OR 0.71, *p* = 0.03) whereas untreated hypertension was no longer significant (OR 0.78, *p* = 0.27), but the effect size was similar.

**Table 4:** Association between risk factors and the presence of hippocampal calcifications

Determinant	Crude OR (95% CI)	*p* value	Adjusted OR* (95% CI)	*p* value	Multivariable OR (95% CI)	*p* value
Age (per decile)	**1.35 (1.23–1.49)**	**.00**	**1.37 (1.24–1.51)**	**.00**	**1.41 (1.26–1.57)**	**.00**
Gender (male)	1.00 (0.78–1.28)	.98	1.13 (0.88–1.46)	.35	1.04 (0.80–1.36)	.80
Stroke in medical history	**1.55 (1.17–2.05)**	**.00**	**1.40 (1.08–1.87)**	**.02**	1.21 (0.88–1.64)	.24
Hypertension	1.13 (0.88–1.45)	.34	0.90 (0.69–1.18)	.44	**0.74 (0.56–0.99)**	**.049**
Diabetes mellitus	**1.86 (1.34–2.59)**	**.00**	**1.68 (1.20–2.35)**	**.00**	**1.57 (1.10–2.25)**	**.01**
Hyperlipidaemia	**1.85 (1.43–2.39)**	**.00**	**1.71 (1.31–2.22)**	**.00**	**1.63 (1.20–2.22)**	**.00**
Smoking						
Current	1.09 (0.79–1.50)	.62	1.35 (0.96–1.91)	.08	1.30 (0.91–1.84)	.15
Former	1.19 (0.87–1.61)	.28	1.17 (0.85–1.61)	.35	1.10 (0.79–1.53)	.59
Never	Reference		Reference		Reference	
Obesity, BMI > 30	0.89 (0.63–1.27)	.47	0.96 (0.67–1.36)	.80	0.85 (0.67–1.40)	.97
eGFR < 60	0.97 (0.68–1.38)	.72	0.71 (0.49–1.03)	.07	0.72 (0.49–1.05)	.09
1st degree family < 60 years with CVD history	1.02 (0.45–1.39)	.99	1.04 (0.75–1.43)	.83	0.99 (0.71–1.39)	.97

*Adjusted for age and gender

*OR* odds ratio, *CVD* cardiovascular disease, *eGFR* estimated glomerular filtration rate [ml/min/1.73 m^2^, = 32,788 × (serum creatinine)^−1.154^ × (age)^−0.203^ × (0.742 if woman) × (1.210 if black)], *BMI* body mass index in kg/m^2^

## Discussion

The main findings of our study are that calcification of the hippocampus is common in a stroke population and increasingly prevalent at higher age. HC was related to several classic cardiovascular atherosclerotic risk factors—age, history of DM and hyperlipidaemia—but not to others, e.g. smoking, male gender, CVD history and renal dysfunction. In multivariate analysis, a weak inverse association with hypertension was found.

From the multivariate analysis, we conclude that HC is independently related to age and history of DM and hyperlipidaemia, all of which have been described as risk factors for cardiovascular events, mortality and arterial calcification in various vascular beds [5]. While older age and DM are related to multiple vascular abnormalities, hyperlipidaemia clearly points to atherosclerosis [6, 7]. Atherosclerosis is a chronic condition of the intimal layer of the vascular wall which involves accumulation of lipids, inflammation and calcification and can cause cardio- and neurovascular events [8]. Arterial calcifications often co-localize with these atherosclerotic intimal lesions and are therefore frequently used as a surrogate marker for atherosclerosis on CT images [9]. Other forms of arterial calcification, such as internal elastic lamina calcification and Mönckeberg sclerosis or medial calcification, are primarily associated with chronic kidney disease, genetic syndromes, older age and DM, but not with inflammation and hyperlipidaemia [10]. Therefore, our epidemiological analysis suggests that HC is vascular in origin and most likely atherosclerotic, although further validation is required. Studies looking into the underlying process for HC with histopathological analysis are scarce. Wegiel et al. examined hippocampi of Alzheimer’s disease (AD), Down syndrome and control patients and described a vasculopathy in the walls of hippocampal (pre)capillaries and arteries that they named ‘vascular fibrosis and calcification’ (VFC) [2]. VFC was found to occur in AD patients and age-matched controls in 59% and 57% respectively and was shown to cause loss of neurons in the CA1 sector, dentate gyrus and subiculum proper through degeneration and occlusion of blood vessels. The most severe form of VFC, progressing to the hippocampal head, appeared to be related to hippocampal sclerosis. Interestingly, Wegiel et al. concluded that VFC is not typical for atherosclerosis and that it appeared to be a type of non-atherosclerotic calcification.

What could be the possible consequences of our findings? There are limited data to suggest that HC, possibly as a marker for vascular disease, is related to cognitive decline [3]. HC was most frequently found in the posterior hippocampus in both CA1 and the dentate gyrus. These hippocampal regions have been related to AD, vascular disease and ageing. HC could therefore play a role in neurodegenerative, vascular and age-related hippocampal disease [11, 12]. A possible relation to AD, however, appears less likely because even though HC was frequently found in patients with AD, Wegiel et al. reported that only 4% of patients with Down syndrome showed VFC while 96% were affected by AD, opposing a common pathological mechanism [2]. While AD and HC might have a combined effect on hippocampal function and cognition, distinction between both pathologies can be of importance to measure preventive and treatment interventions. Risk factors for HC identified in our study could provide a starting point for treatment as both DM and hyperlipidaemia are modifiable: first and foremost through prevention, and secondly through lifestyle improvements like treatment with lipid-lowering drugs, glucose stabilization and exercise. High levels of blood glucose and type 2 DM have been related to metabolic defects within the hippocampus and hippocampal atrophy [12, 13]. The beneficial effects of exercise and fitness on hippocampal volume and cognition have been described and treatment with cholesterol-lowering therapy slowed cognitive decline and atrophy of both medial temporal lobes [14, 15].

The presence and severity of HC in this cohort appears mostly symmetrical, although some dissimilarities are present. HC was found more often unilaterally right, which was also described in other studies, and was on average slightly more severe on that side [3]. The bilateral function of the hippocampus is largely unknown; however, evidence of hippocampal lateralization has been reported [16, 17]. In cases of unilateral hippocampal atrophy both the maintenance as well as the decline of cognitive function has been described [18, 19].

After correcting for other CVD risk factors we found a weak inverse relation of ‘history of hypertension’ with HC even though there was a higher prevalence of HC in these patients. A good explanation here is lacking and it points at the possible limitations of our study. First, the cohort in which this study was performed consisted entirely of patients with (suspected) stroke without a healthy reference sample. This limits the generalizability of the prevalence of HC to the general population and some caution with the risk factors is warranted, especially on the preventative effect of hypertension. Second, HC was scored on thin slice images which are more susceptible to image noise compared to regular/thick slice images. The rationale for this is that subtle HC can easily be missed on 5-mm slices. While the use of thin slice images can increase false positive findings, we are confident that the distinction can be made adequately with the methods described in our study. Third, data from a multicentre study was used with different CT scanners. However, all CT scanners were state-of-the-art with good quality CT scans and we think this did not influence our visual scores. Fourth, HC was scored categorically and not volumetrically. The categorical score was preferred as it is fast and reproducible and volumetric measurement based on a density/HU threshold of 130 could prove difficult because of the adjacent choroid plexus and image noise on thin slice data. Furthermore, dichotomous logistic regression analysis could now be performed, providing odds ratios which are often more understandable for readers/physicians. Fifth, information on BMI and family history were missing for more than 30% of the patients. We imputed the data using multiple imputation to allow for optimal multivariable analysis. Finally, to assess HC in this sizable cohort, it was divided and scored by three observers showing a good interobserver agreement (Fleiss’ kappa 0.81). The advantage of this approach was that we were able to investigate a large cohort with reasonable effort. However, our approach might have introduced some level of error in the prevalence estimation and associations compared to a situation where multiple observers would have scored all images with a subsequent consensus reading to solve discrepancies. On the basis of the blinded analysis and good agreement between observers, the misclassification is likely non-differential and therefore unrelated to the investigated risk factors. While non-differential misclassification can cause overestimation, it often results in an underestimation of the reported associations [20].

To conclude, HC is a frequent finding in a cohort of patients suspected of acute stroke and is related to modifiable cardiovascular risk factors such as hyperlipidaemia and DM. Still, our findings need to be confirmed and further radiology–pathology and radiology–outcome association studies on this subject are needed. This may lead to a new strategy for prevention and treatment of these modifiable risk factors of HC and associated cognitive decline.

## Electronic supplementary material

Supplementary Fig. I (GIF 47 kb)

High resolution image (TIFF 2394 kb)

## Abbreviations

ANOVA: Analysis of variance
CA1: Cornu Ammonis 1
CVD: Cardiovascular disease
DM: Diabetes mellitus
DUST: Dutch acute stroke trial
eGFR: Estimated glomerular filtration rate
HC: Hippocampal calcification
IQR: Interquartile range
OR: Odds ratio
VFC: Vascular fibrosis and calcification

## References

[1] APT C, Gupta G, Alatakis S (2012). Hippocampal calcification prevalence at CT: a retrospective review. Radiology.

[2] Wegiel J, Kuchna I, Wisniewski T (2002). Vascular fibrosis and calcification in the hippocampus in aging, Alzheimer disease, and Down syndrome. Acta Neuropathol.

[3] Kockelkoren R, De Vis JB, Mali WPTM (2016). Hippocampal calcification on computed tomography in relation to cognitive decline in memory clinic patients: a case-control study. PLoS One.

[4] van Seeters T, Biessels GJ, van der Schaaf IC (2014). Prediction of outcome in patients with suspected acute ischaemic stroke with CT perfusion and CT angiography: the Dutch acute stroke trial (DUST) study protocol. BMC Neurol.

[5] Allison MA, Hsi S, Wassel CL (2012). Calcified atherosclerosis in different vascular beds and the risk of mortality. Arterioscler Thromb Vasc Biol.

[6] Lanzer P, Boehm M, Sorribas V (2014). Medial vascular calcification revisited: review and perspectives. Eur Heart J.

[7] Allison MA, Criqui MH, Wright CM (2004). Patterns and risk factors for systemic calcified atherosclerosis. Arterioscler Thromb Vasc Biol.

[8] Weber C, Noels H (2011). Atherosclerosis: current pathogenesis and therapeutic options. Nat Med.

[9] Rumberger JA, Simons DB, Fitzpatrick LA (1995). Coronary artery calcium area by electron-beam computed tomography and coronary atherosclerotic plaque area. Circulation.

[10] Boström K (2016) Where do we stand on vascular calcification? Vascul Pharmacol. 10.1016/j.vph.2016.05.014 10.1016/j.vph.2016.05.014 PMC5097669 27260939

[11] Ashton D, Van Reempts J, Haseldonckx M, Willems R (1989). Dorsal-ventral gradient in vulnerability of CA1 hippocampus to ischemia: a combined histological and electrophysiological study. Brain Res.

[12] Wu W, Brickman AM, Luchsinger J (2008). The brain in the age of old: the hippocampal formation is targeted differentially by diseases of late life. Ann Neurol.

[13] Korf ES, White LR, Scheltens P, Launer LJ (2006). Brain aging in very old men with type 2 diabetes: the Honolulu-Asia Aging Study. Diabetes Care.

[14] Fotuhi M, Do D, Jack C (2012). Modifiable factors that alter the size of the hippocampus with ageing. Nat Rev Neurol.

[15] Tendolkar I, Enajat M, Zwiers MP (2012). One-year cholesterol lowering treatment reduces medial temporal lobe atrophy and memory decline in stroke-free elderly with atrial fibrillation: evidence from a parallel group randomized trial. Int J Geriatr Psychiatry.

[16] Shipton OA, El-Gaby M, Apergis-Schoute J (2014). Left-right dissociation of hippocampal memory processes in mice. Proc Natl Acad Sci.

[17] Zhou H, Zhou Q, Xu L (2016) Unilateral hippocampal inactivation or lesion selectively impairs remote contextual fear memory. Psychopharmacology (Berl). 10.1007/s00213-016-4394-7 10.1007/s00213-016-4394-7 27485536

[18] Schmidt CSM, Lassonde M, Gagnon L (2015). Neuropsychological functioning in children with temporal lobe epilepsy and hippocampal atrophy without mesial temporal sclerosis: A distinct clinical entity?. Epilepsy Behav.

[19] Nedelska Z, Andel R, Laczó J (2012). Spatial navigation impairment is proportional to right hippocampal volume. Proc Natl Acad Sci U S A.

[20] Jurek AM, Greenland S, Maldonado G, Church TR (2005). Proper interpretation of non-differential misclassification effects: expectations vs observations. Int J Epidemiol.

