# Supplementary figures and images for: Hippocampal calcification on brain CT: prevalence and risk factors in a cerebrovascular cohort
Source: Eur Radiol. 2018 Apr 4;28(9):3811–8. doi: 10.1007/s00330-018-5372-8 (PMC6096610; doi:10.1007/s00330-018-5372-8)

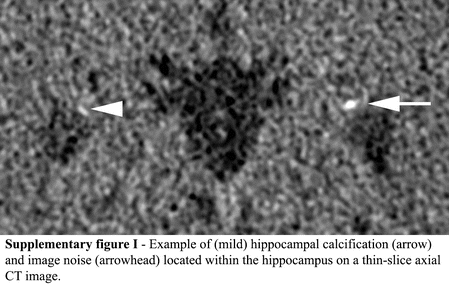

Supplement: Supplementary file 1 — (GIF 47 kb) [file 330_2018_5372_Fig3_ESM.gif]

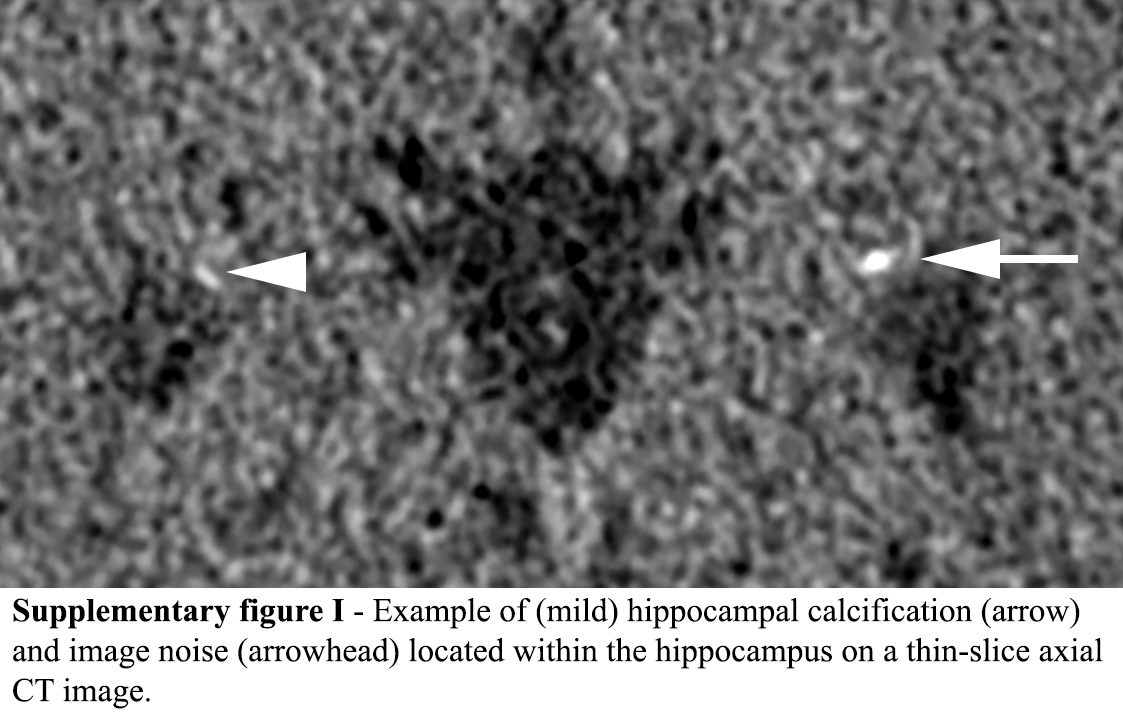

Supplement: Supplementary file 2 — High resolution image (TIFF 2394 kb) [file 330_2018_5372_MOESM1_ESM.tif]
